# The WHO's who of haematopoietic malignancies

**DOI:** 10.1038/sj.bjc.6605230

**Published:** 2009-08-11

**Authors:** S H Oram, B Gottgens

**Affiliations:** 1 Department of Haematology, University of Cambridge, Cambridge Institute for Medical Research, Hills Road, Cambridge CB2 0XY, UK

A revised edition of the core diagnostic text, *Classification of Tumours of Haematopoietic and Lymphoid Tissues*, has recently been published by the World Health Organization (WHO) in collaboration with the European Association for Haematopathology, the Society for Hematopathology and a panel of 30 internationally renowned scientists and haematologists. Haematological neoplasms were first included in the Third Edition of the WHO series on histological and genetic typing of human tumours in 2001 and heralded a significant change in practice and a much-needed harmonisation of what was becoming an increasingly complex myriad of different diagnostic classification systems. This new edition includes the diagnostic criteria for newly characterised neoplasms and incorporates revised information for previously classified neoplasms in light of recently identified additional scientific and clinical knowledge. As in the previous edition, the data are presented in a strictly disease-oriented manner and clinical information, diagnostic criteria, pathological features and associated recurrent genetic aberrations are detailed.

The WHO classification of lymphoid neoplasms was initially largely based on the Revised European–American Lymphoma Classification (REAL), the most widely used monograph before 2001. In the revision, due credence has been given to the fact that the presence or absence of certain translocations in B lymphoblastic leukaemia/lymphoma carries such prognostic significance that it is now appropriate to subdivide this entity based largely on specific recurring chromosomal anomalies into seven distinct subgroups, with an eighth group for those remaining cases not meeting the defining criteria for any of the seven other subgroups.

The WHO classification of myeloid neoplasms integrated ideas from a variety of sources, including the French–American–British Cooperative Group classification (FAB) and the Polycythaemia Vera Study Group (PVSG), and also added a number of novel concepts and classifications, including a lowering of the required blast percentage threshold to diagnose acute myeloid leukaemia from 30% to 20% and the creation of four defined subgroups of acute myeloid leukaemia: (i) acute myeloid leukaemia with recurrent genetic abnormalities, (ii) acute myeloid leukaemia with multilineage dysplasia (now renamed acute myeloid leukaemia with myelodysplasia-related changes), (iii) therapy-related acute myeloid leukaemia and myelodysplastic syndrome, and (iv) acute myeloid leukaemia not otherwise categorised for those cases not meeting the criteria for any of the other subgroups. The new edition includes further revision and refinement in light of an increasing body of research identifying prognostic significance to new and previously identified recurrent genetic anomalies. By the addition of more recurrent cytogenetic anomalies to the list, the group of cases that would previously have fallen into the ‘not otherwise categorised’ subgroup has reduced significantly. In recognition of the fact that the body of literature regarding the significance of certain genetic aberrations is not yet mature, provisional entries have also been featured. There has been clarification of leukaemias with features of both myeloid and lymphoid leukaemia in that a new category of ‘acute leukaemia of ambiguous lineage’ has been added and the defining criteria for diagnosis of what would have previously been called bilineal or biphenotypic leukaemia have been altered. Similarly, there has been renaming and refinement of the classification of myelodysplastic syndromes in the new edition; the presence of ring sideroblasts is no longer considered to be significant in the presence of multilineage dysplasia, refractory cytopaenia with unilineage dysplasia replaces the host of cytopaenic-lineage-specific diagnoses and a provisional entry of ‘refractory cytopaenia of childhood’ has also been entered. A new category of ‘myeloid/lymphoid neoplasms with eosinophilia and PDGRFB rearrangement’ has been created within the myeloproliferative/myelodyplastic disorders classification and significant changes have been made within the myeloproliferative disorders diagnostic classification (now termed myeloproliferative neoplasms in recognition of the malignant nature of these disorders). Appropriate emphasis has been placed on the presence of the JAK2 V617F and similar activating mutations in parallel with recent clinical practice. Additional clinical and laboratory criteria are also offered to facilitate diagnosis and subclassification.

With the publication of the previous edition of the WHO Classification of Tumours of Haematopoietic and Lymphoid Tissues, the provision of an authoritative, concise reference book provided an international standard for haematologists and histopathologists. This finally permitted easy comparison of data between countries and continents, and the book quickly became an invaluable manual both in routine clinical practice and when designing studies to monitor response to treatment and clinical outcome. The refinements in the new edition reflect the fact that for any classification system to remain up to date and relevant, a regular account of recently published, high-quality, peer-reviewed data needs to be given. By carefully fine-tuning the diagnostic criteria for those categories previously recognised and considered, and by creating diagnostic standards for those conditions recently identified, it will be possible to diagnose patients more accurately, design trials looking at outcomes in a less heterogeneous population and ultimately, therefore, facilitate a greater understanding of the molecular basis for these conditions. In all, it is an invaluable text for scientists, pathologists and clinicians alike.

